# FGF21 Promotes Endothelial Cell Angiogenesis through a Dynamin-2 and Rab5 Dependent Pathway

**DOI:** 10.1371/journal.pone.0098130

**Published:** 2014-05-21

**Authors:** Usman Yaqoob, Kumaravelu Jagavelu, Uday Shergill, Thiago de Assuncao, Sheng Cao, Vijay H. Shah

**Affiliations:** Gastroenterology Research Unit, Mayo Clinic, Rochester, Minnesota, United States of America; Ohio State University, United States of America

## Abstract

Binding of angiogenic molecules with cognate receptor tyrosine kinases (RTK) is required for angiogenesis however the precise link between RTK binding, endocytosis, and signaling requires further investigation. Here, we use FGFR1 as a model to test the effects of the large GTPase and endocytosis regulatory molecule dynamin-2 on angiogenic signaling in context of distinct FGF ligands. *In vitro*, overexpression of dominant negative dynamin-2 (DynK44A) attenuates FGFR1 activation of Erk and tubulogenesis by FGF2. Furthermore, we identify FGF21, a non-classical, FGF ligand implicated in diverse human pathologies as an angiogenic molecule acting through FGFR1 and β-Klotho coreceptor. Disruption of FGFR1 activation of ERK by FGF21 is achieved by perturbation of the function of both dynamin-2 and Rab5 GTPase. *In vivo*, mice harboring endothelial selective overexpression of DynK44A, show impaired angiogenesis in response to FGF21. In conclusion, dynamin dependent endocytosis of FGFR1 is required for *in vitro* and *in vivo* angiogenesis in response to FGF2 and the non-classical FGF ligand, FGF21. These studies extend our understanding of the relationships between RTK binding, internalization, endosomal targeting, and angiogenic signaling.

## Introduction

Angiogenesis defines the formation of new vessels from existing vasculature. To achieve angiogenesis, endothelial cells (EC) undergo anatomic changes that facilitate migration, proliferation, and other processes that culminate in vascular sprouting, lumen formation, and branching morphogenesis [1]. Receptor tyrosine kinases (RTK) play an integral role in this process making the understanding by which these proteins function an area of major significance [2].

The fibroblast growth factor (FGF) receptor-1 (FGFR1) family of RTKs is essential for angiogenesis [3], [4]. FGF ligand binding with FGFR1 results in receptor dimerization, phosphorylation, and recruitment of adaptor proteins that culminate in angiogenic signal transduction [3], [4], [5]. Classical FGF ligands act in an autocrine or paracrine manner to bind FGFR1 owing to heparin binding domains that facilitate bioavailability and binding with FGFR1 [6], [7], [8]. However, signaling through FGFR1 can be achieved through other FGF ligands [9], [10]. Unlike classical FGFs, non-classical FGFs lack affinity for heparin binding and require Klotho family member proteins as co-receptors to bind and activate FGFR1 [5]. For example FGF21 binds FGFR1 through co-receptor function of β-Klotho [11] and has been implicated in a liver injury pathway that can lead to organ fibrosis and associated angiogenesis [12]. The link between FGFR1 angiogenesis and fibrosis is further buttressed by recent studies showing EC selective deletion of FGFR1 makes mice resistant to liver fibrosis [13]. These studies highlight the need for better understanding of non-classical FGF ligands for angiogenesis and associated disease pathologies.

Several steps of the RTK endocytosis pathway have been previously characterized. Upon ligand binding, the RTK/ligand complex undergoes endocytosis from the plasma membrane though either adaptor protein-mediated clustering of receptors within clathrin-coated pits or through lipid raft enriched, flask shaped vesicles termed caveolae [14], [15], [16]. Budded and internalized vesicles are targeted for fusion with early endosomes, then subsequently for lysosomal degradation, or alternatively for plasma membrane recycling [17]. However, the precise relationship of distinct vesicle trafficking steps with signal transduction remain an area of active investigation, especially in context of angiogenic signaling through FGFR1 in which prior studies have revealed varying outcomes [18], [19].

Dynamin-2 is a large GTPase responsible for the scission of newly formed vesicles at the plasma membrane, a step that is required for endocytosis [20], [21], [22], [23]. Current models implicate the GTPase activity of dynamin as the driver by which vesicles are pinched off from the plasma membrane. A point mutation construct at lysine 44 of dynamin-2 (DynK44A) disrupts GTPase activity and is commonly used to experimentally probe dynamin endocytic functions [17]. Prior studies have implicated dynamin in EC survival and *in vitro* tubulogenesis through regulation of multiple angiogenic signaling pathways including VEGF and nitric oxide [22]. However, evidence that dynamin and its GTPase activity regulate angiogenesis *in vivo* is lacking.

Here, we explore the link between the endocytosis and signaling of RTKs using FGFR1 as a prototype. We identify a requisite role for dynamin dependent endocytosis of FGFR1 and its endosomal targeting, for optimal FGFR1 angiogenic signaling. Similar effects are observed in response to perturbation of Rab 5 function. Additionally, we provide *in vivo* evidence for the role of dynamin-2 GTPase function in angiogenesis using newly generated transgenic mice over-expressing the dominant negative dynamin-2 K44A, which display impaired angiogenesis. This is detected in response to both the classical ligand, FGF2 and even more importantly, in response to the non-classical ligand, FGF21. In total, the work identifies a key role for dynamin dependent endocytosis in growth factor signaling *in vitro* and *in vivo* and uncovers a new role for FGF21 in angiogenesis.

## Materials and Methods

### Cell culture and transfection

EC used in these studies included human liver endothelial cells (LEC) (ScienCell, San Diego, CA), freshly isolated murine LEC and human umbilical vein endothelial cells (HUVEC, purchased from Lonza Inc, Allendale, NJ). Cells were grown in EC growth medium containing 5% fetal bovine serum, 2% endothelial cell growth supplement and 1% penicillin/streptomycin (ScienCell, San Diego, CA) and maintained in standard tissue culture conditions (37°C, 5% CO_2_ incubator). Adenoviral, retroviral and lentiviral transduction was performed as described previously [22]. Adenoviral vectors were generated through the Iowa Vector Core and encoded DynK44A, or LacZ, control. FGFR-1-flag, β-Klotho-flag, and Rab5-DN-myc adenoviral vectors were generated using AdEASY as described previously [24]. Cells were incubated for 1 hour with 0.1% albumin/PBS with 50 MOI of adenoviruses which achieved transduction efficiency approximating 90% with minimal toxicity. For siRNA transfection, scrambled control or FGFR1 (Qiagen) was transfected into EC by using Oligofectamine Reagent (Invitrogen, Carlsbad, CA). All assays were performed 72–96 hours after transfection. Protein knockdown by siRNA was confirmed by Western blot analysis.

### Biotin labeling, Immunoprecipitation, and Western Blot

For biotin labeling, serum-starved EC were labeled with biotin using 20 mM solution of NHS-biotin reagent (EZ link NHS-SS-Biotin Thermo Scientific) in PBS for 30 minutes at 4°C. Cells were then washed with PBS and incubated with growth factor at 37°C for 30 minutes to stimulate endocytosis. Cells were washed with 50 mM reduced glutathione (GSH, 75 mM NaCl, 75 mM NaOH, pH 8.0) on ice to remove surface biotin then lysed in modified radioimmune precipitation assay buffer. After centrifugation, the cell lysate was incubated with streptavidin-conjugated agarose beads (Sigma) to pull down biotinylated proteins. After washing with lysis buffer, the protein complex of streptavidin-conjugated agarose beads with biotinylated protein was eluted with 2× sample buffer. Samples were analyzed by SDS-PAGE and Western blotting to detect FGFR1 and β-Klotho. For Western blotting, cells were lysed in RIPA buffer, fortified with protease inhibitor (Roche) and clarified. Protein extracts were subjected to denaturing 12% SDS-polyacrylamide gels and transferred to nitrocellulose membranes. After blocking, blots were probed with antibodies when included anti-V5, anti-Dynamin-2, ERK, pAKT ser473, total ERK, total AKT, flag, pFGFR 653/654, pFGFR 766, pFGFR 400, FGFR1, β-actin (Cell Signaling, BD Transduction Laboratories, Invitrogen, Abcam and Sigma). After secondary antibody incubation, antigen-antibody complexes were detected by enhanced chemiluminescence detection system (ECL Plus, Santa Cruz). Equal protein loading was verified by re-probing the membrane with an anti-β-actin antibody (1∶5000).

### Transferrin uptake assay

To evaluate clathrin-mediated endocytosis, the internalization of fluorescent labeled transferrin was performed as previously described [25]. Briefly primary murine LEC were isolated as previously described [26] and incubated in EC growth medium containing 2% fat-depleted BSA (Sigma) followed by incubation with 10 µg/ml Alexafluor 488 conjugated transferrin (Molecular Probes) for 15 min at 37°C. Cells were washed and photographed using confocal microscope (Zeiss LSM Pascal Axiovert; Carl Zeiss Ltd.). Transferrin positive signal was quantified using MetaMorph Software (version 7.6, Molecular Devices, USA).

### Generation of DynK44A^fl/fl^ mice

Transgenic mice were generated with endothelial-specific over-expression of dynamin-2 K44A (DynK44A^fl/fl^). This mutation imparts a defect in the GTPase activity of dynamin-2 [22]. DynK44A dominant negative transgene was constructed by inserting the ∼2.8-kb cDNA encoding the entire ORF for rat dynamin-2 mutated at site 44 from K to A into a Bgl II site in a previously constructed plasmid, that contained chicken alpha actin promoter, simian virus 40 (SV40) intron poly (A) signal and IRES that drives GFP expression to yield pOL9-CAC-DynK44A-GFP, which will be described further in Results. The construct was linearized and pronuclear injections were done by the Mayo Transgenic facility. Originally, 3 founder lines were established (DynK44A 132, 51 and 112) and two of these were phenotypically analyzed in detail in this study. Second generation animals 2–5 months of age were used for all studies. Offspring were screened and genotyped by PCR for the presence of the V5 tag and the floxed sequence. Transgenic DynK44A mice were subsequently crossed with Tie2-Cre mice [27] to generate endothelial specific expression of DynK44A (TIE2^Cre^/DynK44A^fl/fl^) which were also used in depicted studies as indicated. All animal experiments were approved by the Mayo Clinic Institutional Care and Use Committee (IACUC) and carried out in accordance with institutional guidelines. Our IACUC committee approved our animal protocol number A10213 for use in this study. All surgeries were performed using appropriate anesthesia and analgesia (Ketamine/Xylazine) along with Bacitracin Zinc antibiotic at the suture site to help prevent infection, and all efforts were made to minimize suffering.

### *In vitro* 3D fibrin gel bead assay

The fibrin gel bead assay was performed as described previously [28]. Human LEC were transduced with an adenovirus vector encoding LacZ or dynamin dominant negative mutant, K44A. Transduced cells were allowed to attach to the Cytodex-3 microcarrier beads (GE Healthcare) by incubating in complete EC medium for 4 hours at 37°C with repetitive inversion. Cell-coated beads were embedded in a fibrin and thrombin matrix (500 beads per ml) and were incubated at 37°C in a humidified 5% CO_2_ incubator and the medium was replaced daily for 7 days. Endothelial outgrowth images were captured using an inverted microscope (Zeiss Axiovert 40 CFL, 4× magnification), and quantified using Image pro software.

### Aortic ring assay

Aortic ring explant cultures were performed as previously described [26]. Excised and 1-mm cut murine aortic rings were placed in 100 µl of Matrigel (growth factor reduced; BD Biosciences (Cat. No 356231). Rings were cultured in endothelial cell medium and incubated at 37°C in a humidified 5% CO_2_ incubator with medium replacement daily for 7 days. Rings were incubated in media with varying compounds and adenoviruses (10^9^ PFU) as indicated in specific experiments. When micro-vessels began to sprout, rings were fixed in 4% paraformaldehyde. Photographs of rings were captured using a phase contrast microscope (Zeiss, 10× magnification). Morphometric analysis of CD31 positive cell sprouting specifically within the vessel ring lumen was quantified using Image Pro Software (Bethesda, MD).

### Matrigel plug assay

*In vivo* angiogenesis was performed by injecting sterile Matrigel (growth factor reduced; BD Biosciences) into the subcutaneous layer of anesthetized mice. Matrigel plugs were removed 7 days after implantation and photographed. Hemoglobin content was determined by Drabkin method according to the manufacturers protocol (Sigma, St. Louis, MO) and absorbance measured at 540 nm. The concentration of hemoglobin was calculated and normalized to the plug weight.

### Ear angiogenesis assay

Ear angiogenesis experiments were performed as described previously [29]. 8 to 10-week male mice were injected subcutaneously in ear with an adenovirus vector encoding Cre recombinase (AdCre; 10^9^ PFU in 20 µl) subcutaneously and as a control AdGFP was injected in contralateral ear. After 6 days, mice were sacrificed and auricles were removed and embedded in OCT for frozen sectioning (5 µm). Sections were immunostained for vWF and quantified for vWF positive structures in a blinded fashion.

### Directed *in vivo* Angiogenesis Assay (DIVAA)

Directed *in vivo* angiogenesis assay was performed using growth factor reduced basement membrane extract. Briefly silicon tubes of 1.5 cm length were closed at one end and prefilled with extract with and without growth factors (FGF2 and FGF21) and allowed to solidify at 37°C for 1 hour. Silicon tubes were implanted subcutaneously in mice. After two weeks, mice were sacrificed and tubes were recovered and imaged using a Leica MZ125 microscope. The extract was further analyzed by hemoglobin quantitation and harvested by paraffin embedding for H&E staining.

### FGF analysis from human liver cirrhosis specimen

Normal and cirrhotic human liver samples obtained from biopsy and/or surgical waste under the Mayo Clinic Institutional Review Board (IRB)-approved protocols (Protocol Number 10-005760) were used for Western blot analysis, quantitative PCR (qPCR) and/or immunostaining studies.

### Statistical analysis

Results are expressed as mean ± SEM from at least three independent experiments. Two-tailed Student' T-test or ANOVA was used to test the statistical significance between the groups as appropriate. A P-value of less than 0.05 was considered as statistically significant.

## Results

### FGFR1 activation of ERK by FGF2 and 21 is blocked by Dyn-K44A

**Figure 1:**
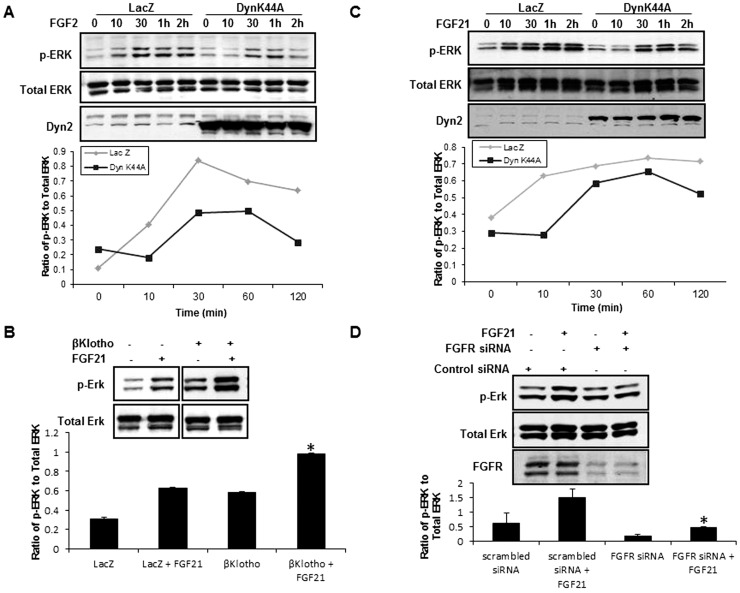
DynK44A abrogates FGF activation of ERK in EC. A. EC were incubated with FGF2 for the time period indicated. Cell lysates were analyzed by Western blotting for pERK and total ERK. Overexpression of DynK44A is depicted in the lower panel. Densitometric analyses of Western blots are shown in the graph. B. β-Klotho or LacZ were overexpressed in EC using adenovirus and then cells were treated with FGF21 and analyzed by Western blot for pERK and total ERK. Lower panel shows densitometric analysis from Western blots from three independent experiments (*p<0.05). C. EC overexpressing LacZ or β-Klotho were stimulated with FGF21 for the time period indicated and analyzed by Western blot for pERK and total ERK. DynK44A expression is shown in the lower panel with densitometric analysis shown in the graph. D. FGFR1 was knocked down in EC by siRNA and cells were treated with FGF21 and analyzed by Western blot with densitometric analysis shown in the graph (n = 3, *p<0.05).

FGF2 ligand engagement with FGFR1 represents a canonical RTK angiogenic activation pathway in EC which we used as a model to study how dynamin dependent endocytosis regulates RTK signaling [18]. We used the phosphorylation of ERK, a signaling molecule downstream from FGFR1 as a prototypic readout to ascertain FGFR1 signaling [19]. Upon stimulation with FGF2, FGFR1 activates ERK and the duration and intensity of ERK activation is attenuated by overexpression of DynK44A (Figure 1A). However, signaling through FGFR1 can be achieved through other FGF ligands [9]. One of these, FGF21, is a member of the FGF19 subfamily; FGF19, 21 and 23. FGF21 has been studied in context of liver injury and other disease processes [12] but its effects on EC and angiogenesis have not been investigated in depth. The FGF21 ligand is selectively dependent on the β-Klotho isoform, a divergent structural member of the glycosidase I superfamily [30]. EC expression of β-Klotho is markedly reduced in response to culturing of cells (Figure S1A, B) [31]. Owing to this phenomenon and so as to easily track the β-Klotho/FGFR1 complex, we used an adenoviral expression system to overexpress β-Klotho-flag tagged construct for our FGF21 related studies. FGF21 stimulation of EC leads to ERK activation which is further enhanced in the presence of adenovirally overexpressed β-Klotho (Figure 1B). DynK44A again abrogates FGF21 dependent activation of ERK (Figure 1C). Furthermore, FGF21 signaling in EC is dependent on FGFR1 as EC with FGFR1 knockdown by siRNA are unable to activate ERK (Figure 1D). Thus, these studies indicate that FGFR1 signal activation by FGF2 and FGF21 requires dynamin dependent receptor endocytosis.

### FGF ligand activation of ERK requires targeting of FGFR1 to early endosomes

**Figure 2:**
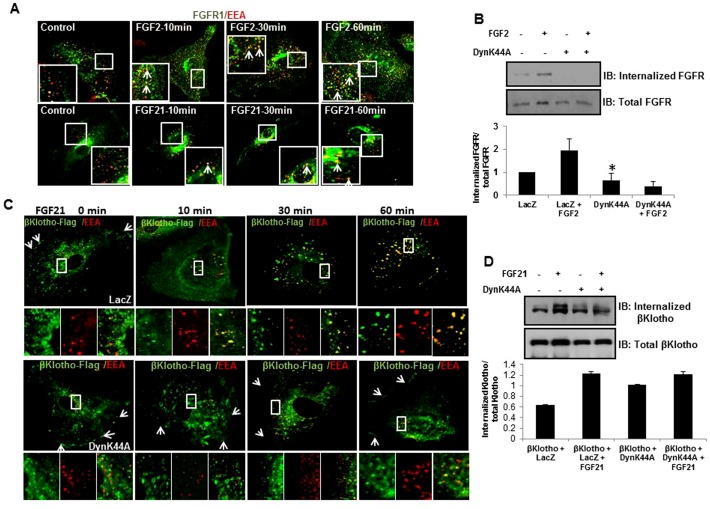
DynK44A blocks FGF2 and FGF21 stimulated internalization of FGFR1 and β-Klotho. A. Confocal images of EC with FGF2 and FGF21 stimulation over time period indicated were immunostained with FGFR1 (green) and EEA (Early Endosomal Antigen) (red) respectively (upper panel). Zoomed images indicate colocalization of FGFR1 and EEA (yellow) (n = 3, *p<0.05). B. EC with and without overexpression of DynK44A, were labeled with biotin and stimulated with FGF2 and analyzed by Agarose Streptavidin pull-down for FGFR1 internalization as depicted in the immunoblot. Total FGFR1 is shown in the lower panel. Densitometric analysis of the Western blots was done from three independent experiments (*p<0.05). C. Immunofluorescence staining of EC is shown with adenoviral overexpression of β-Klotho-flag and DynK44A and stimulation with FGF21 using β-Klotho-flag (green) and EEA (red) antibody. Arrows indicate fluorescence signal of the receptor at the plasma membrane. Zoomed images focus on β-Klotho receptor complex colocalization with EEA. D. Biotinylation studies for receptor internalization were done using EC with overexpression of β-Klotho-flag and DynK44A. Western blots were analyzed for internalized β-Klotho upon FGF21 treatment. Lower panel shows total protein levels of β-Klotho.

First, to determine the subcellular itinerary of FGFR1 upon ligand stimulation, we performed immunofluorescence studies which show that FGF2 and FGF21 incubation with cells results in colocalization of FGFR1 with EEA1, an early endosome marker (Figure 2A). Surface biotinylation studies for receptor internalization confirm that FGFR1 is internalized in response to FGF2 stimulation and also show that overexpression of DynK44A abrogates the internalization of FGFR1 stimulated by FGF2 (Figure 2B).

We next examined receptor internalization in the FGF21/FGFR1/β-Klotho signaling model. EC with adenoviral transduction of β-Klotho-Flag tag were immunostained for flag and EEA1 antibodies. This analysis revealed reduction in the membrane distribution of β-Klotho-flag which was used to track its coreceptor partner FGFR1 upon FGF21 stimulation (Figure 2C). Colocalization of β-Klotho-flag staining with EEA1 was evident by confocal microscopy (Figure 2C). Endocytosis inhibition by overexpression of DynK44A abrogated β-Klotho endocytosis (Figure 2C). These results were corroborated by biotinylation studies (Figure 2D) which in total indicate that FGF21 activation of ERK requires endocytosis of β-Klotho coreceptor in tandem with FGFR1 (Figure 2D).

### FGFR1 targeting to early endosomes and activation of ERK requires Rab5

**Figure 3:**
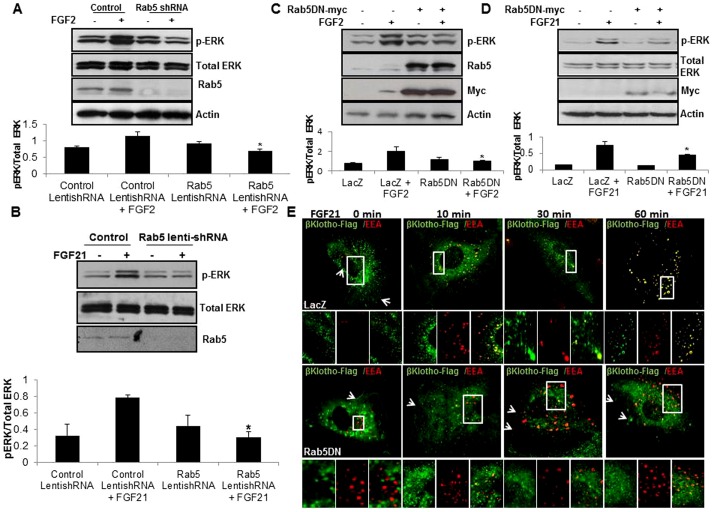
FGF stimulated ERK activation is abrogated by disruption of Rab5 function. A. EC were transfected with Rab5 lenti-shRNA to knockdown Rab5 and then stimulated with FGF2. Protein lysates were analyzed for ERK activation by Western blot. Rab5 knockdown is shown in the blot in the upper panel while quantitation is shown graphically below (n = 3, *p<0.05). B. EC with lentiviral overexpression of β-Klotho and Rab5 shRNA were stimulated with FGF21 and analyzed for ERK activation by Western blotting. Representative blot and quantitation of densitometry from multiple experiments is shown (n = 3, *p<0.05). C. EC with adenoviral overexpression of Rab5DN-myc construct were analyzed for ERK activation by Western blot. Rab5 and myc-protein levels are shown in the middle panels while quantitation of densitometry from multiple experiments is shown below (n = 3, *p<0.05). D. EC with Rab5 DN-myc construct overexpression were stimulated with FGF21. Cell lysates were subjected to Western blot analysis with indicated antibodies to detect ERK activation and overexpression of Rab5. Quantitation of densitometry from multiple experiments is shown (n = 3, *p<0.05). E. Confocal images including zoomed images are shown of EC with overexpression of β-Klotho-flag and Rab5 DN-myc, stimulated with FGF21 over indicated time period and stained with Flag and EEA antibodies; arrows indicate receptor fluorescence signal at the plasma membrane.

The role of receptor endocytosis in signaling is an area of active investigation. For example, internalization of receptors may lead to receptor degradation, receptor recycling, or endosomal signal activation [32]. Rab GTPase proteins are critical for specific endosomal sorting steps with Rab5 regulating early endosome formation [33]. Therefore, we further interrogated the effects of Rab5 perturbation on FGFR1 signaling. First, we utilized a Rab5 shRNA to verify whether inhibition of receptor internalization and early endosome targeting attenuates FGFR1 mediated ERK activation. Knockdown of Rab5 using lenti shRNA in EC abrogated FGFR1 signaling upon FGF2 stimulation (Figure 3A). Similarly FGF21 stimulation of FGFR1 is also blocked by depletion of Rab5 suggesting that FGF21 signaling requires membrane internalization and early endosomal targeting of FGFR1 (Figure 3B). Three different clones of Rab5 lenti-shRNA were tested for specificity and showed similar effects (Figure S2A, B). We examined this model further with a dominant negative Rab5 construct, generated by point mutation of serine 34 to aspartate in the Rab GTP binding domain [34]. FGFR1 mediated ERK activation in response to FGF2 and 21 was blocked by adenoviral overexpression of Rab5 DN-Myc tag in EC (Figure 3C, D). FGF21 mediated endocytosis of β-Klotho receptor complex was also blocked by Rab5 DN in EC as assessed by confocal microscopy (Figure 3E). These data indicate that Rab5 dependent early endosomal targeting is required for maximal FGFR1 activation of ERK.

### FGF21 promotes angiogenesis *in vitro* and *in vivo*

**Figure 4:**
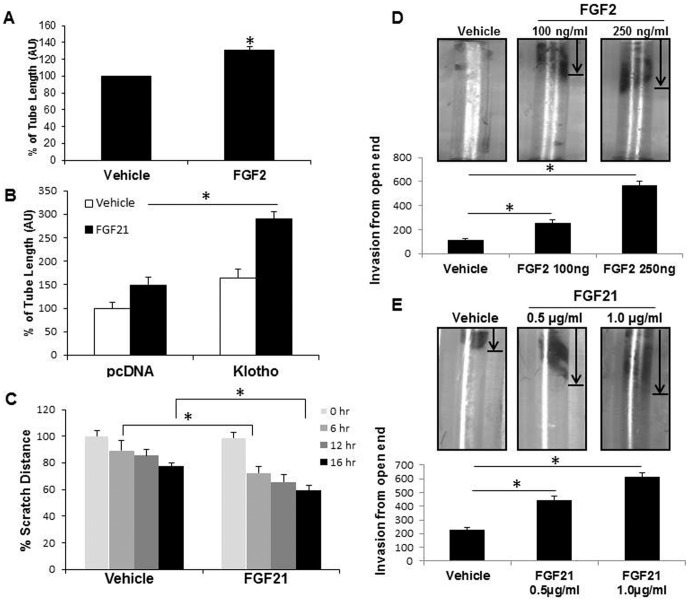
FGF21 promotes angiogenesis *in vitro* and *in vivo*. A. EC were plated on growth factor reduced Matrigel and stimulated with FGF2 and tubulogenesis was measured after 6(n = 3, *p<0.05). B. EC with and without overexpression of β-Klotho were plated on Matrigel and treated with FGF21 and analyzed after 6 hours for tube formation (n = 3, *p<0.05). C. Confluent monolayer of EC with βKotho overexpression were scratched and treated with FGF21 and analyzed for cell migration by measuring width of scratch. (n = 3, *p<0.05). D and E. Directed *in vivo* angiogenesis assay was carried out in mice using growth factor reduced basement membrane extract with and without FGF2 (D) and FGF21 (E). The silicon tubes were implanted subcutaneously. After two weeks, mice were sacrificed and tubes recovered and analyzed for angiogenesis by imaging using Leica MZ125 microscope. Vascular invasion was quantified using MetaMorph Imaging software (n = 3, *p<0.05).

We next transferred our analysis of FGFR1 activation of ERK to an angiogenesis read-out. First, we examined if FGF21, like FGF2, could promote angiogenesis. FGF2 promotes angiogenesis in EC in an FGFR1 dependent manner as evidenced by increased tube formation in the presence of FGF2 (Figure 4A). FGF21 also promotes tube formation in EC and this effect is enhanced with overexpression of β-Klotho (Figure 4B). An alternative *in vitro* angiogenic read-out, the scratch assay also reveals increased migratory capacity of EC with β-Klotho overexpression upon FGF21 stimulation (Figure 4C). Based on angiogenic function *in vitro*, we then tested FGF21 for *in vivo* angiogenesis using a directed *in vivo* angiogenesis assay (DIVAA). In this assay, basement membrane extract premixed with and without angiogenic factors in a semiclosed silicon cylinder is implanted subcutaneously into the mice. Like FGF2, FGF21 promotes angiogenesis in a dose dependent fashion (Figures 4D, E). These results show that FGF21 is an angiogenic ligand *in vitro* and *in vivo*.

### FGFR1 induced angiogenesis is attenuated in transgenic mice over-expressing DynK44A

**Figure 5:**
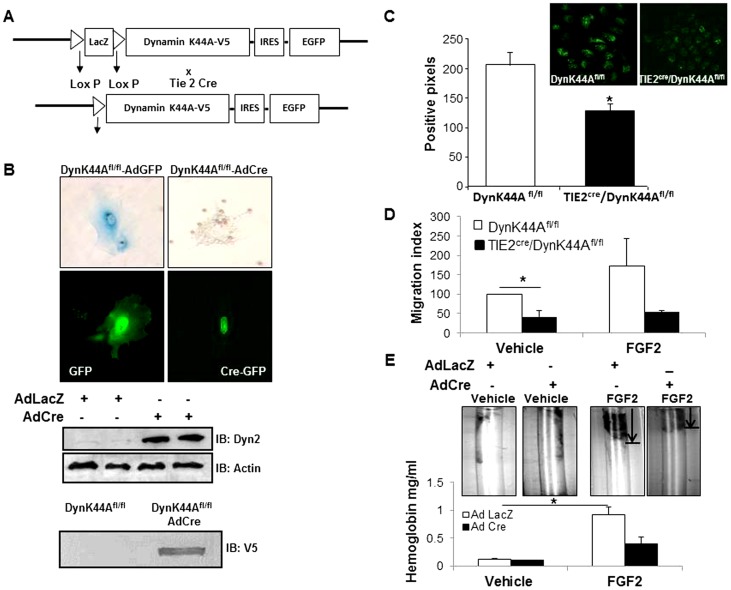
Overexpression of DynK44A in EC impairs migration and endocytosis. A. Strategy for generation of mouse with genetic overexpression of DynK44A in EC is shown. Cre recombinase mediated LacZ gene deletion leads to overexpression of DynK44A in EC from DynK44A^fl/fl^ mice. B. LEC isolated from DynK44A^fl/fl^ mouse were transduced with Ad GFP or Ad Cre-eGFP and stained for X-gal to detect LacZ and images were captured. Confocal images for GFP and Cre-GFP are shown in the lower panel. Immunoblots are shown in the lower panel for DynK44A overexpression in response to AdCre transduction of mouse LEC. C. LEC were isolated from TIE2Cre/DynK44A^fl/fl^ mice and incubated with 10 µg/ml Alexafluor 488 conjugated transferrin for 15 min at 37°C. Cells were photographed using confocal microscope. Transferrin positive signal was quantified using MetaMorph Software in the lower panel (*p<0.05). D. Transwell cell migration was carried out with isolated mouse LEC treated with FGF2 and graph was plotted showing the number of cells migrated. E. Images from directed *in vivo* angiogenesis assay are shown. Basement membrane extract was premixed with AdCre and Ad LacZ adenovirus along with FGF2 and filled into silicon tubes before subcutaneous implantation in DynK44A^fl/fl^ mice. Tubes were recovered from mice after two weeks of implantation and analyzed for hemoglobin quantitation as depicted (*p<0.05).

Since inhibition of endocytosis and endosomal targeting of FGFR1 attenuates ERK activation in EC stimulated with FGF2 and FGF21, we next examined effects of dynamin inhibition on FGFR1 dependent angiogenesis. Prior studies have demonstrated that dynamin-2 positively regulates diverse angiogenic pathways in EC although evidence to support a role for dynamin in angiogenesis *in vivo* is lacking [22], [23]. To address this gap, we generated a transgenic mouse that contains a floxed stop codon that precedes a dynamin-2 gene containing a point mutation at DynK44A^fl/fl^. These mice were crossed with a mouse line in which Cre was driven by the TIE-2 gene or alternatively cells were isolated from DynK44A^fl/fl^ floxed mice for analysis (Figure 5A). To confirm the expression of DynK44A, liver EC were isolated from Founder 1 (F1) pups from two different founder lines and transduced with AdCre. Both lines evidence transgenic protein expression as determined by V5-immunoblot from isolated liver EC (Figure 5B, bottom). ECs from mice stained for X-gal demonstrate deletion of LacZ from mice transduced with AdCre (Figure 5B, top). The DynK44A^fl/fl^ mice also show Cre mediated overexpression of DynK44A in EC as demonstrated by immunoblotting (Figure 5B, middle). Furthermore, as anticipated with overexpression of K44A, these EC had less uptake of transferrin as compared to EC from WT mice (Figure 5C). EC isolated from DynK44A^fl/fl^ mice also fail to migrate in the presence of FGF2 as compared to EC isolated from WT mice (Figure 5D), indicating that these mice provide an appropriate *in vivo* model to explore FGF signaling. Therefore we used DynK44A^fl/fl^ mice for DIVAA to further investigate angiogenic effects of FGF ligands. AdCre or AdLacZ were added to basement membrane extract along with FGF2 to assess effects of DynK44A on EC invasion. FGF2 promotes angiogenesis in the AdLacZ group while this effect is reduced in the AdCre group as assessed by hemoglobin estimation (Figure 5E).

**Figure 6:**
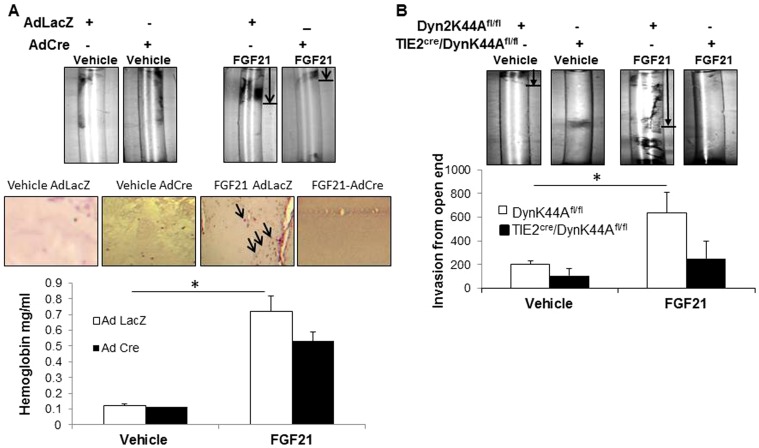
DynK44A blocks FGF21 mediated angiogenesis. A. Basement membrane extracts were premixed with AdCre or Ad LacZ adenovirus along with FGF21 and filled into the silicon tubes before subcutaneous implantation in DynK44A^fl/fl^ mice. 2 weeks after implantation, mice were sacrificed and tubes were recovered, and analyzed for hemoglobin quantitation. Images of basement membrane extract from directed *in vivo* angiogenesis, H&E stain from paraffin sections of extract recovered from mouse implants, and hemoglobin quantitation are all shown (*p<0.05). Arrows indicate vascularity. B. Basement membrane extracts were premixed with FGF21, filled into the silicon tubes and implanted in TIE2^cre^/DynK44A^fl/fl^ or DynK44A^fl/fl^ mice. Vascular invasion was quantified using MetaMorph Imaging software images from directed *in vivo* angiogenesis are shown (n = 4, *p<0.05).

We next examined the angiogenic effect of FGF21 in DynK44A^fl/fl^ mice using DIVAA. We used AdCre and AdLacZ virus with FGF21 or vehicle in the basement membrane extract to test specifically the effects of DynK44A on FGF2 angiogenesis. Extracts were evaluated for vascular invasion and hemoglobin estimation as measures of angiogenesis. Like FGF2, FGF21 promotes angiogenesis as assessed by hemoglobin quantitation in mice received AdLacZ (Figure 6A). However, mice that received AdCre with FGF21 demonstrate less angiogenesis as evidenced by reduction in hemoglobin content in basement membrane extracts. We then used TIE2^cre^/DynK44A^fl/fl^ mice with EC specific overexpression of DynK44A in our *in vivo* angiogenesis studies. The angiogenic effect of FGF21 was abrogated in TIE2^cre^/DynK44A^fl/fl^ mice compared to control mice (Figure 6B). These data show that FGF21 is an angiogenic ligand and that its signaling in EC *in vivo* is blocked by DynK44A. It is important to note that DynK44A^fl/fl^ mice and AdDynK44A evidence an anti-angiogenic phenotype in a variety of *in vivo* and *in vitro* angiogenic models independent of direct FGF stimulation as well (Figure S3).

### Upregulation of FGFR1 signaling molecules in human liver cirrhosis

**Figure 7:**
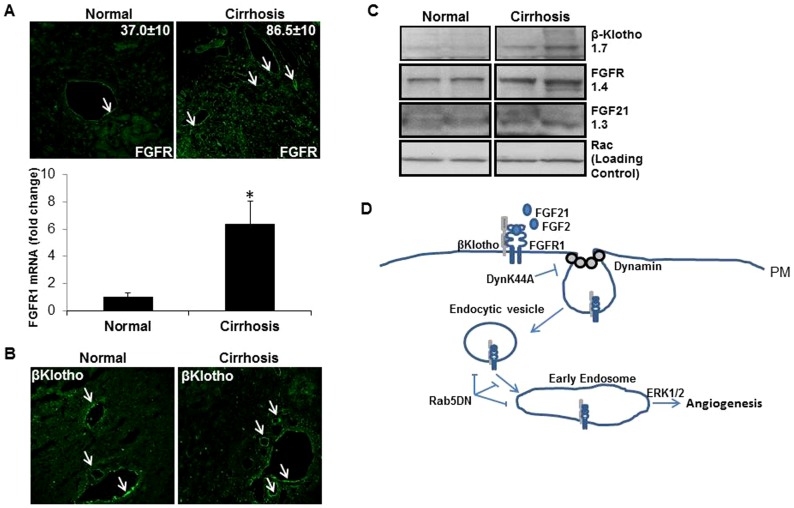
Upregulation of FGFR1 pathway in human liver cirrhosis and its mechanistic role in a proposed endocytosis regulated model of FGFR1 signaling. A. Nonalcoholic steatohepatitis associated human liver cirrhosis shows increased FGFR1 immunostaining (green) compared to normal human liver (quantitation from multiple samples is shown in each representative image). Lower panel shows increased FGFR1 mRNA levels from cirrhotic liver tissue by qRT-PCR (*p<0.05). B. Confocal images of β-Klotho immunostaining on liver sections from normal and cirrhosis patients are shown (arrows). C. Western blot shows significant increase in FGFR1, Klotho and FGF21 levels from human cirrhotic liver samples compared to control (2 normal and 2 cirrhosis are shown). Depicted values represent fold change in cirrhotic samples compared to control by densitometry. C. FGF21 stimulation of FGFR1 leads to activation of ERK and internalization of FGFR1 and β-Klotho co-receptor. Internalization of FGFR1 upon ligand stimulation requires dynamin dependent endocytosis. ERK activation is abrogated by perturbation of Rab5 function as well indicating importance of early endosomal targeting of FGFR1. Thus, full ERK activation and angiogenesis by FGF2 and FGF21 requires FGFR1 internalization and FGFR1 endosomal targeting by dynamin-2 and Rab 5, respectively.

Finally, we sought to examine protein levels of FGFR1, FGF21 and β-Klotho in a human disease condition associated with angiogenesis. To this end, human samples from controls or patients with liver cirrhosis from nonalcoholic steatohepatitis were assayed since cirrhosis is associated with angiogenesis [35], and since FGF21 has been previously implicated in steatohepatitis liver injury pathways [12]. We studied the expression levels of FGFR1 in liver from normal and cirrhosis patients by immunostaining and qPCR. Confocal images from normal and cirrhotic human liver sections showed increased liver EC staining of FGFR1 within fibrous septa (Figure 7A) which was confirmed by real-time qPCR (Figure 7A, lower panel). Next we examined β-Klotho expression in liver sections from normal and cirrhotic patients through immunostaining. In addition to diffuse parenchymal hepatocyte staining, β-Klotho immunostaining was prominently detected within liver EC based on co-localization of β-Klotho with the EC marker protein, vWF (Figure 7B, Figure S4A). Western blot analysis confirms increased FGFR1 levels as well as increased levels of β-Klotho and FGF21 in human cirrhotic liver compared to normal liver (Figure 7C). A similar increase in β-Klotho mRNA levels was observed by RT-PCR from liver tissue procured from mice with experimental liver injury (chronic carbon tetrachloride administration) [36] (Figure S4B). These translational studies provide evidence of human applicability to the aforementioned mechanistic studies.

## Discussion

Angiogenesis contributes to diverse physiological and pathobiological processes including wound healing, tumor microenvironment, and others, with FGF signaling playing a seminal role in this process. Indeed, FGFR1 signal transduction is one of the most important angiogenic pathways in EC. In this study, we make a number of novel observations including 1) the FGF21 ligand promotes angiogenesis *in vivo* and *in vitro*, 2) FGFR1 activation and angiogenesis by FGF21 requires the β-Klotho co-receptor and its internalization, 3) FGFR1 activation of angiogenic signaling requires internalization of plasma membrane FGFR1, and 4) perturbation of endocytosis and early endosomal targeting disrupts FGFR1 angiogenesis *in vivo*. Our proposed model of FGF21 signaling in EC is depicted schematically in Figure 7D. Thus, the present study expands our knowledge of FGF mediated angiogenic signaling.

Membrane bound receptors such as FGFR1 are thought to be internalized into early endosomes upon ligand binding [37], however subsequent vesicle sorting steps and signaling are not well understood. For example, while some internalized receptors are returned to the plasma membrane through recycling endosomes, other receptor pools are sorted to late endosomes for eventual lysosomal degradation [38]. Furthermore, the specific compartment in which FGFR1 resides that is responsible for downstream signal activation is incompletely defined. Some work suggests that FGFR1 receptors signal exclusively at the plasma membrane while more recent studies indicate that signal initiation requires receptor compartmentalization into intracellular endosomes [18], [39]. Effects of dynamin depletion using siRNA, genetic deletion and dynamin GTPase inhibitor have led to mixed results with regards to internalization of RTK such as FGFR1 and VEGFR [18], [40], [41], [42]. This may be due in part to different endocytic pathways that mediate internalization of different RTK. For example, in a recent study in endothelial cells, FGFR1 internalization was shown to occur through a macropinocytic pathway while other studies have also shown that FGFR1 is internalized by clathrin, caveolin or lipid raft mediated endocytosis [18], [43], [44], [45], [46], [47]. Since prior publications have focused on dynamin-2 siRNA approach, we focused on a dominant negative approach in this work using a novel genetic modification that leads to K44A overexpression selectively in EC. We show that dynamin K44A overexpression attenuates labeled FGFR1 internalization indicating the importance of dynamin-2 GTPase activity in FGFR1 internalization. It is possible that some of these differences may be due to divergent effects of dominant negative GTPase inhibition and siRNA based dynamin depletion. As DynK44A may block both plasma membrane vesicle internalization as well as subsequent intracellular vesicle sorting steps, we next evaluated effects of perturbation of Rab5 to dissect these possibilities further, as discussed below.

Rab proteins partner with motor proteins to stimulate vesicle trafficking by tethering and guiding vesicles [48], [49]. Their mechanistic roles are relatively selective for distinct endosomal sorting steps. It is well established that Rab5 regulates early endosomal structure [33]. We explored this through complementary knock down and dominant negative loss of function approaches targeting Rab5 [50]. Indeed, the Rab5 mutant as well as Rab5 knock-down decreased ERK phosphorylation in response to FGFR1 activation. Biotinylation and microscopic studies showed that FGFR1 internalization and endosomal sorting was disrupted by perturbation of Rab5 function. These data suggest that FGFR1 endosomal targeting is required for optimal activation of ERK.

While prior studies focused on an alternative angiogenic kinase VEGFR2, have suggested that endocytosis is also required for full activation of ERK [51]; other studies have revealed divergent observations [52]. In the present study, time course stimulation of EC with FGF2 showed that full activation of ERK requires endosomal signaling since ERK activation was blocked by DynK44A without effects on phosphorylation of FGFR1. This was further corroborated with Rab5 perturbation studies which also attenuated ERK activation downstream from FGF2 engagement. Thus, dynamin and its endocytic regulatory function appears critical for FGFR1 function.

Emerging evidence implicates dynamin-2 dependent endocytosis in angiogenesis [22], [23]. Dynamin-2 has been implicated in EC survival and subsequent tubulogenesis through NO dependent S-nitrosylation [22], [53]. In EC, dynamin GTPase activity has been thought to promote angiogenesis through effects on vesicle trafficking relevant to the function of KDR, eNOS, and other angiogenic proteins [22], [23]. Our *in vitro* studies showed that endocytosis of FGFR1 and its co-receptor Klotho can be stimulated by FGF ligand in EC and is blocked by DynK44A. However, the role of dynamin-2 for *in vivo* angiogenesis has remained elusive, owing to the embryonic lethality of the dynamin-2 global knockout mouse [54]. To explore this *in vivo*, we generated a mouse with endothelial selective overexpression of DynK44A to explore the endocytic pathway of FGFR1 and its role in angiogenesis upon FGF ligand stimulation. Mice overexpressing EC selective DynK44A demonstrated impaired angiogenesis as evidenced by reduced endothelial aortic sprouts, ear angiogenesis, reduced endothelial matrigel invasion and cell migration in response to FGF ligand stimulation. Thus, disruption of dynamin-2 GTPase function impairs angiogenesis both *in vitro* and *in vivo*.

While canonical FGF ligands bind FGFR1 by virtue of heparin-binding affinity, nonclassical FGF ligands including FGF21 require co-receptors for engagement with FGFR1. Klotho is a membrane protein that is related to β-glucuronidases, and acts as a co-receptor for engagement of FGF21 with FGFR1 [30]. As FGF21/FGFR1 engagement is best characterized for metabolic functions, we show here a new and important role for FGF21 and β-Klotho for angiogenesis. Our data demonstrates that FGF21 induces FGFR1 internalization from the plasma membrane reminiscent of that observed with FGF2 ligand. Inhibition of this process by DynK44A blocks ERK phosphorylation that occurs downstream from FGFR1. Interestingly, prior studies found raised serum FGF21 levels in patients with non-alcoholic fatty liver disease, a metabolic liver disease supporting further investigation of this ligand in the pathogenesis of liver disease [55]. Since angiogenesis is now well recognized to accompany liver injury, inflammation, and fibrosis [56], FGF21 release from injured, fat-laden parenchymal cells may stimulate liver EC angiogenesis, thereby providing a link, supported by our own human data as well, between metabolic liver injury and angiogenesis. In addition to potential therapeutic links of FGF21 to glucose and lipid metabolism [57], its role as a potential biomarker for metabolic and other forms of liver injury warrant further investigation.

In summary, FGF21 is a novel angiogenic ligand that signals through FGFR1 and β-Klotho in EC to activate ERK, which promotes angiogenesis *in vitro* and *in vivo*. Dynamin-dependent endocytosis is required for full activation of ERK by FGF21 and is dependent on β-Klotho and FGFR1. This work should have significance for broad biological functions and disease processes ranging from metabolism, liver injury, and angiogenesis.

## Supporting Information

Figure S1 **β-Klotho expression is lost upon in vitro culture of EC.** A. Real time PCR from isolated mouse LEC was done to examine β-Klotho mRNA levels before and after culture. B. Western blot analysis from isolated mouse liver EC both freshly isolated and after culture was done to examine the protein levels of β-Klotho.
(TIF)

Figure S2 **Rab5 knockdown in HUVEC abrogates ERK activation upon FGF 2 and FGF21 stimulation.** A. HUVEC transduced with three distinct clones of Rab5 lenti-shRNA were treated with FGF2 after overnight serum starvation. Lysates were collected for Western blot and membrane was probed for pERK. Actin and total ERK controls are shown. B. 3 distinct clones of Rab5 lenti-shRNA were used to transfect HUVEC and then treated with FGF21. Lysates were collected for Western blot using pERK antibody. Total ERK and actin controls are shown.
(TIF)

Figure S3 **DynK44A overexpression in mice leads to reduced EC sprout formation and angiogenesis.** A. 3D fibrin gel bead assay was performed using human liver EC transduced with adenovirus vector encoding LacZ or DynK44A. After 7 days, EC outgrowth images were captured and quantified using Image Pro software (upper panel) (*p<0.05). Lower panel, cells were stained with F-actin binding reagent Phalloidin for 1 hour to show the decreased filipodia structures in DynK44A transduced cells. B. Images of Matrigel plugs were removed 7 days after subcutaneous implantation into mice. Hemoglobin content was determined by Drabkin method with absorbance measured at 540 nm, normalized to plug weight, and graphed (*p<0.05). C. Confocal images from vWF staining of mouse ear injected with control virus and AdCre. Morphometric analysis of vWF positive staining was done and is depicted in the graph. D. Phase contrast images of aortic ring explants from TIE2cre/DynK44Afl/fl and DynK44Afl/fl control mice in Matrigel incubated in EC medium for a week. Representative sprouts (upper panel) and quantitation are shown. Dynasore (30 µM; dynamin inhibitor) was used as a positive control (*p<0.05) (FCS-fetal calf serum).
(TIF)

Figure S4 **β-Klotho colocalizes with vWF in human liver cirrhosis and its levels are elevated in a murine model of liver injury.** A. Liver sections from normal and cirrhotic patients were co-immunostained using β-Klotho and vWF antibodies. β-Klotho and vWF colocalized in the EC lining of the vessels within the liver as indicated by arrows. B. RT-PCR analysis was done from liver tissue of mice treated with CCL4 showing increase β-Klotho in these mice compared to vehicle (olive oil) (n = 3 from each group *p<0.05).
(TIF)
