# Supplementary figures and images for: FGF21 Promotes Endothelial Cell Angiogenesis through a Dynamin-2 and Rab5 Dependent Pathway
Source: PLoS One. 2014 May 21;9(5):e98130. doi: 10.1371/journal.pone.0098130 (PMC4029959; doi:10.1371/journal.pone.0098130)

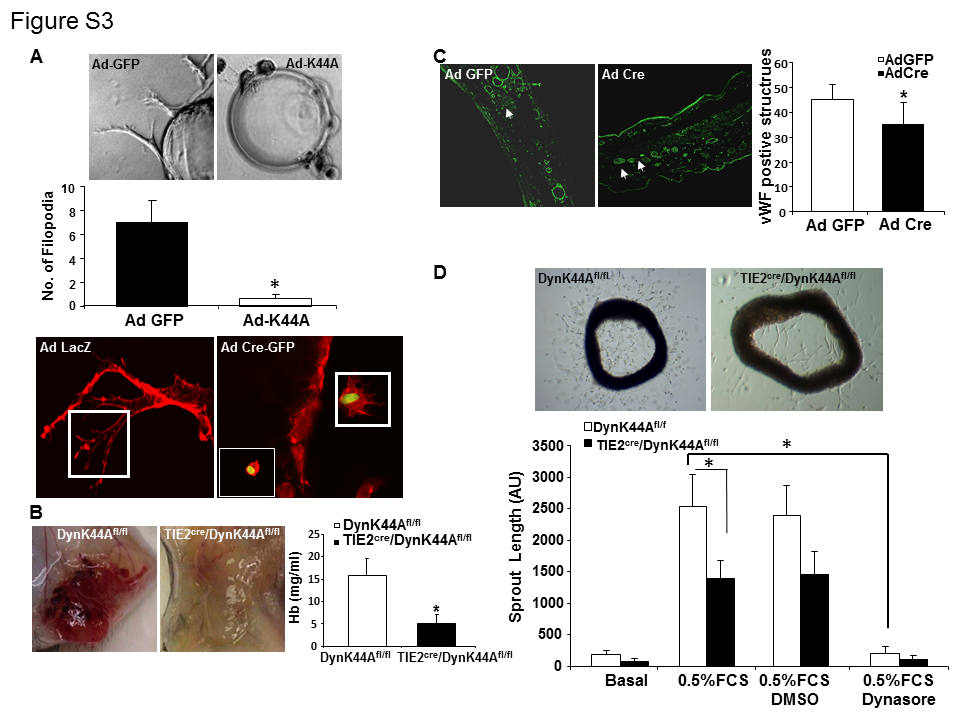

Supplement: Figure S3 — DynK44A overexpression in mice leads to reduced EC sprout formation and angiogenesis. A. 3D fibrin gel bead assay was performed using human liver EC transduced with adenovirus vector encoding LacZ or DynK44A. After 7 days, EC outgrowth images were captured and quantified using Image Pro software (upper panel) (*p<0.05). Lower panel, cells were stained with F-actin binding reagent Phalloidin for 1 hour to show the decreased filipodia structures in DynK44A transduced cells. B. Images of Matrigel plugs were removed 7 days after subcutaneous implantation into mice. Hemoglobin content was determined by Drabkin method with absorbance measured at 540 nm, normalized to plug weight, and graphed (*p<0.05). C. Confocal images from vWF staining of mouse ear injected with control virus and AdCre. Morphometric analysis of vWF positive staining was done and is depicted in the graph. D. Phase contrast images of aortic ring explants from TIE2cre/DynK44Afl/fl and DynK44Afl/fl control mice in Matrigel incubated in EC medium for a week. Representative sprouts (upper panel) and quantitation are shown. Dynasore (30 µM; dynamin inhibitor) was used as a positive control (*p<0.05) (FCS-fetal calf serum). (TIF) [file pone.0098130.s003.tif]

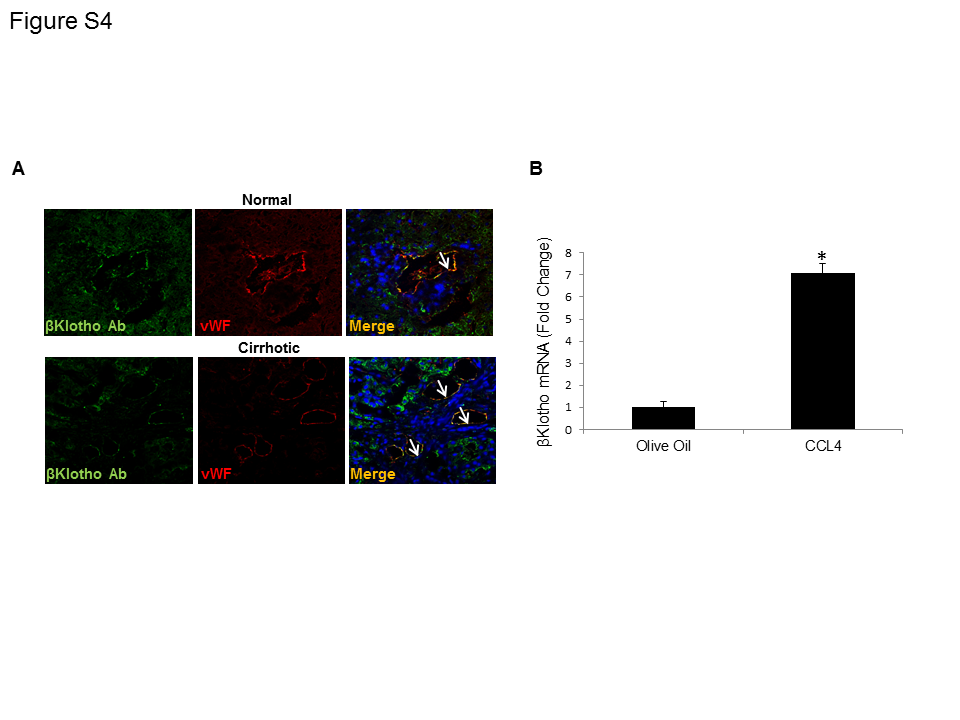

Supplement: Figure S4 — β-Klotho colocalizes with vWF in human liver cirrhosis and its levels are elevated in a murine model of liver injury. A. Liver sections from normal and cirrhotic patients were co-immunostained using β-Klotho and vWF antibodies. β-Klotho and vWF colocalized in the EC lining of the vessels within the liver as indicated by arrows. B. RT-PCR analysis was done from liver tissue of mice treated with CCL4 showing increase β-Klotho in these mice compared to vehicle (olive oil) (n = 3 from each group *p<0.05). (TIF) [file pone.0098130.s004.tif]
